# Current status, challenges and prospects for dairy goat production in the Americas

**DOI:** 10.5713/ajas.19.0256

**Published:** 2019-07-01

**Authors:** Christopher D. Lu, Beth A. Miller

**Affiliations:** 1 College of Agriculture, Forestry and Natural Resource Management, University of Hawaii, Hilo, HI 96720, USA; 2 Department of Natural and Physical Sciences, University of Arkansas - Pulaski Technical College, North Little Rock, AR 72118, USA

**Keywords:** Dairy Goats, Americas, Goat Milk, Production Efficiency, Environment, Animal Welfare

## Abstract

Dairy goat production continues to be a socially, economically and culturally important part of the livestock industry in North, Central and South America and the Caribbean islands. Goat milk, cheese and other dairy products offer consumers food products with nutritional, health and environmental benefits. In North America, Mexico produces the greatest volume of goat milk, but most is for family or local consumption that is typical of a mixed farming system adopted by subsistence farmers in dry areas. The United States is not yet a large global goat milk producer, but the sector has expanded rapidly, with dairy goat numbers doubling between 1997 and 2012. The number of dairy goats has also increased dramatically in Canada. Commercial farms are increasingly important, driven by rising demand for good quality and locally sourced goat cheese. In South America, Brazil has the most developed dairy goat industry that includes government assistance to small-scale producers and low-income households. As of 2017, FAO identified Haiti, Peru, Jamaica, and Bolivia as having important goat milk production in the Western Hemisphere. For subsistence goat producers in the Americas on marginal land without prior history of chemical usage, organic dairy goat production can be a viable alternative for income generation, with sufficient transportation, sanitation and marketing initiatives. Production efficiency, greenhouse gas emission, waste disposal, and animal welfare are important challenges for dairy goat producers in the Americas.

## INTRODUCTION

Goat milk is an increasingly important dairy product around the world, with total production increasing from 12 million tonnes in 1993 to nearly 19 million tonnes in 2017 [1]. Although the Western Hemisphere is not considered a major producer, dairy goat products are increasingly consumed and traded. North and South America contributed 4.4% of global production during 2006 through 2017 [1]. In 2017, Asia produced 57%, Africa 24%, and Europe 15% of the global goat milk supply. Historically, goat milk was preferred to cow’s milk only by those allergic to cow’s milk, or for alleviating gastrointestinal disorders because of differences in its physic-chemical characteristics, in the United States and Canada [2]. The “Latin” American countries were colonized by the Spanish or Portuguese who introduced dairy goat breeds and use of their products, resulting in a preference for the taste of goat milk in many areas. Consumers in the Americas are increasingly aware of the nutritional merits of goat milk [3]. Along with an increased appreciation for French style soft goat cheese, and a philosophical preference for locally sourced food, affluent urban consumers are driving the increase in goat milk production.

Domestic goats are not indigenous to the Americas. European breeds were introduced to North America in the 1590s by the Spanish during the colonial period, and are still the most popular breeds. The Swiss dairy breeds are especially common. Nubian goats from Egypt (via England), and Nigerian Dwarf goats from West Africa were imported later, and are also quite common, as are crosses, grade and “criollo” or goats that have adapted to local conditions over the past 500 years. Alpine, LaMancha, Nigerian Dwarf, Nubian, Oberhasli, Saanen, Sable, and Toggenburg are the dairy breeds recognized by the American Dairy Goat Association [4].

In the Latin American countries, Mexico, most of South America and some Caribbean countries, dairy goats make an important contribution to smallholder livelihoods, especially in arid areas that are poorly suited for agricultural crops. Mexico and Brazil are the main dairy goat-producing countries, with small-scale production and local consumption. The United States and Canada have smaller but increasing numbers of dairy goats, and the sector is commercializing rapidly. Agricultural markets are less controlled in North America compared to Europe, and because the dairy goat sector is small, government support is not available as for dairy cow producers. Subsidies and price supports exist for crops such as soybeans and corn used for feed, but prices for goat milk are less regulated. However, demand is strong so prices are higher compared to cow milk.

There are 8.7 million goats in Mexico, 2.6 million in the United States and 0. 2 million in Canada, which may seem insignificant by Asian standards, but all three countries are seeing overall numbers rise, with dairy goats generating significant interest and investment [1]. In the United States and Canada, the goat milk sector has been increasing rapidly since the 1980s. Commercial farms are increasingly important, driven by rising demand for good quality and locally sourced goat cheese. Many small goat dairies produce artisanal cheese, yogurt and other products on the farm, but larger processors are becoming more common. Mass produced goat cheese can be found in supermarkets in all cities and even small towns across North America.

**Figure 1:**
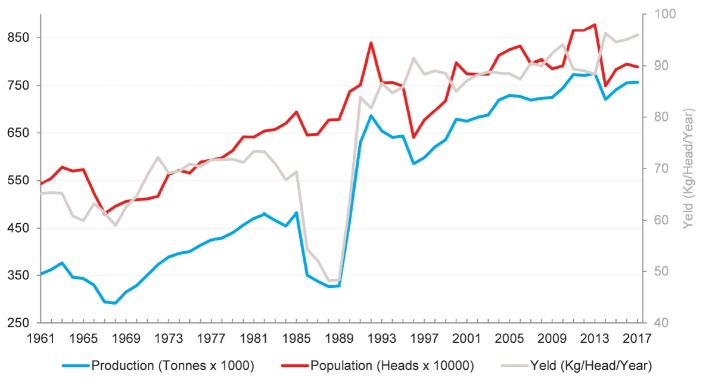
Dairy goat population, total goat milk production and yield per animal in Americas 1961 through 2017 (Compiled from [1], aggregate, may include official, semi-official, estimated or calculated data).

Goat milk production more than doubled while population increased by 46% during 1961 through 2017, resulting in a 47% increase in yield (kg/head/yr) (Figure 1). During the past decade (2007 through 2017) goat milk production increased by 4.9% in Americas while the dairy goat population slightly decreased overall by 0.7% [5]. Continuous productivity improvement is expected with greater commercialization, especially in the United States and Canada, as technical advances in genetics, feeding and management become more widely adopted.

## MEXICO

### Current status

With the largest goat population in the Americas, Mexico’s rural communities continue to rely on goat production economically and culturally. Dairy goat production is one of the three prominent goat production systems in Mexico, along with “cabrito” (sucking kids normally sold for meat at about 10 kg) and “chivo cebado” (meat from mature goats sold at 40 to 45 kg). These three production systems often overlap. While intensive dairy goat operations are found near the larger cities, most dairy goat producers rely on extensive feeding that can be impacted by seasonal rainfall and forage fluctuation. Dairy goat production in dry parts of Mexico tends to be on marginal or degraded land, without adequate management of natural resources [6].

**Figure 2:**
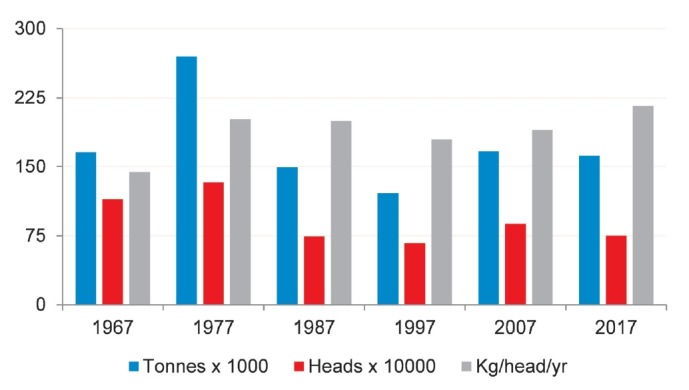
Trends of goat milk production in Mexico (Compiled from [1], may include official, semi-official, estimated or calculated data).

Total goat population increased into the 1980s and then declined until recently (Figure 2). The total number of dairy goats was estimated to be 752,970 in 2017, down from 880,000 a decade earlier [1]. However, goat milk production was on an upward trend since the 1990s, from a low of 120,528 tonnes in 1997 to over 162,323 tonnes in 2017. The yield per animal rose steadily since 1997, with a 20% increase during the past two decades. It implies an increase in production efficiency from improvements in nutrition, breeding and management. Based on these indicators, the dairy goat industry in Mexico is moving towards producing more with fewer animals, which lowers the environmental load. The production and consumption of milk in Mexico increased dramatically from just over 2 million tonnes in the 1960s to nearly 12 million tonnes in the 2010s [1]. It underscores the increasing importance of commercial goat milk production as part of the total supply of milk to a growing urban population. More than 1% of global goat milk is produced in Mexico, slightly higher than her share of world goat meat production.

### Challenges

Mexican consumers appreciate goat milk and products such as “cajete” or goat milk candy, so that demand is strong. However, most goats are raised in dry or marginal areas with minimal management, so production remains quite low. Commercial goat dairies are increasing near urban centers, but challenges include endemic goat diseases such as *Brucella mellitensis* and *Chlamydia* spp., and limited veterinary, extension or laboratory services.

Because of the high nutrient demands for milk output, the availability and cost of feed resources constrain the dairy goat industry in Mexico. Feeds and supplements in an intensive system with stall feeding can be prohibitively expensive. A semi-intensive system combining natural grazing with feed supplements reduces feed cost, but seasonal fluctuation of forage availability can be a challenge. Dairy producers who rely only on unimproved pastures often have low milk production from unmet nutritional requirements.

Synchronization of milk supply with demand is another challenge. Peak milk production occurs in the spring while the highest demand for milk and cheese occurs in the fall/winter. While this challenge is not unique to Mexico, it exacerbates the precarious economic situation of subsistence producers who rely on milk income and frustrates consumers who prefer dairy products from goats.

Because many dairy producers are on marginal land in the semi-arid regions of Mexico, they are generally socio-economically disadvantaged and marginalized from government services. They need adequate income from milk production to improve their standard of living, but are constrained by the productivity of the land. An increase in productivity and income is best achieved through strong producer organizations, technical training, access to credit and financial services, and more organized markets.

### Prospects

Diversification of feed resources can help meet the high nutrient demand for milk production for producers in Mexico. Utilization of crop residues and agro-industrial byproducts has been long recognized as a cost effective alternative for nutrient supply to goat producers [7]. For semi- and intensive dairy producers, substitution of costly grains with agro-industrial byproducts has the potential to reduce feed costs or supply needed feeds during the dry season when pasture is limited. This feeding strategy has gained recognition for nutrient recycling and waste disposal. This is especially important for the smaller dairy producers without the resources to purchase more expensive grains as protein or energy supplements [8–10]. Many of these agro-industrial byproducts can be mixed or ensiled to increase palatability and undesired feeding characteristics and improve feeding values. Nutritional supplementation by small dairy goat producers can optimize milk production in semiarid range environments [11], but such training is usually limited to NGOs or pilot projects. Once production increases, it takes further effort to market the products, often far from the farm.

Manipulation and management for out-of-season breeding in dairy goats in temperate climates is practiced in technically advanced goat dairies when the cost is justified by a favorable market return. As in other countries, producers can induce heat through introduction of a buck, manipulation of light cycles and the administration of exogenous gonadotrophins following progestogen priming with either vaginal sponges or subcutaneous implants. Light treatment, melatonin, and breed affected the outcome of out-of-season breeding in goats [12] and present opportunities for out-of-season breeding in Mexico.

Organic milk production can be promising for producers on marginal land [13]. It can be productive and sustainable, but more susceptible to seasonal fluctuation in forage availability [14]. Small dairy goat producers on marginal land have the opportunity to convert to organic operations as the land generally was not contaminated with chemicals and synthetic fertilizers previously [15]. Small scale producers need considerable guidance to achieve organic certification but can gain additional income from the premium price for organic products, especially if exported to US markets, which largely relies on imports. Organic goat products are in demand because they are perceived to improve animal welfare, protect the environment, and sustain rewarding rural lifestyles [15].

## THE UNITED STATES

### Current status

**Figure 3:**
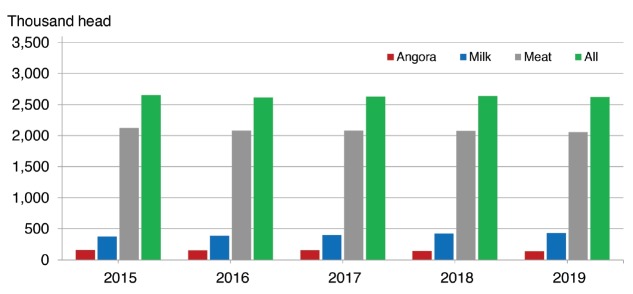
All goats and kids inventory by class in the United States [16].

Out of the estimated 2.6 million goats in 2018 in the USA, about 380,000 (16%) are thought to be raised primarily as dairy animals (Figure 3). Only 12% were identified as dairy goats in 2012, so the trend is upwards.

Currently, the United States does not have a good estimate of total dairy goats, their production or the market behavior in different parts of the country. Goat statistics are still relatively new to the National Agricultural Statistics Service (NASS), with the first ever full-scale goat survey conducted in January 2005. Previous data did not clearly separate the sheep from the goats, the commercial from the pet, or the dairy from the meat animals. Data collection is complicated by multipurpose goats, complex production systems, direct sales of goats to consumers with no passage through a traditional concentration point (auction or slaughter plant) [17].

All 50 states have at least one goat breeders’ association, and there are a plethora of national organizations, websites, magazines, fairs and innovative products such as new goat cheeses, candy and cosmetic products made from goat milk [18]. Nearly half of dairy goat operations (43.5 percent) belonged to a national or local goat association or club [19]. However, these are all private organizations with no government support, and most are quite small with limited impact.

States with the highest numbers of milk goats are Wisconsin, California, Iowa, Texas and Pennsylvania, which are also in the top 12 for dairy cows [20], and are also the states where goat processing factories operate. Dairy goat production benefits from a well-developed dairy cow industry with good agricultural research and extension, as well as supply chains for machinery, feeds, medicine, and vaccines, and a clear and enforced dairy regulatory framework. For the market in dairy goat products to expand, regulations need to be adapted, and strong producer organizations are necessary.

Rising demand is driving the increase in dairy goat production. Immigrants from Mexico, Asia, Africa and the Middle East are leading the demand for goat milk and yogurt. Affluent consumers prefer the health benefits of goat products, or appreciate the taste of French style goat cheeses. The “locovore movement”, connecting food producers and food consumers in the same geographic regions in order to develop more self-reliant and resilient food networks, encourages consumers to purchase and consume foods from small scale producers to build social stability, and enhance environmental stewardship by decreasing transport costs. Many small goat farms are located around major metropolitan areas along the East Coast where wealthier consumers are more likely to appreciate goat cheese and to hold “locovore” views.

### Challenges

In the United States, the regulations for production, processing, and marketing of milk to protect public health are described in the federal Food and Drug Administration (FDA) publication called the Grade A Pasteurized Milk Ordinance (PMO). Each state health department establishes minimum regulations for grade A milk from these standards and may adopt more stringent standards than those of the PMO. The regulations were developed for the dairy cow industry, creating a difficult barrier when goat milk producers began applying for licenses in the 1980s. In 2006, the Dairy Practices Council published the “Guidelines for the Production and Regulation of Quality Dairy Goat Milk” which allowed the sector to flourish within a legal framework [21].

There are significant differences in the national standards for cow and goat milk. In 1991, the minimum somatic cell count (SCC) for “grade A” (highest quality) cow milk was set 750,000 cells/mL, but milk from healthy goats will test much higher, so the 2006 Guidelines permitted an SCC of 1 million cells/mL. This was raised to 1.5 million SCC for goat milk in 2009, making it possible for more producers to market a “grade A” goat milk product, which commands the highest price. Fluid goat milk for consumption or processing is now standardized to ensure the uniformity and legality of finished dairy goat products. These uniform standards have allowed the market in dairy goat products to expand [22], and consumers have come to expect high quality products from healthy animals in sanitary conditions.

Raw or unpasteurized goat milk sales are controversial in the United States, where some consumers strongly believe in its benefits. States may adopt their own laws on raw milk sales. However, at the federal level, the US FDA bans the interstate sale or distribution of raw milk. All milk sold across state lines must be pasteurized and meet the standards of the US PMO [23].

Recording and using production data to improve dairy goat management distinguishes professional or modern goat keeping from traditional or low input strategies. In the United States, official testing and record keeping is done by the Dairy Herd Improvement Association (DHIA), a national and state program of milk testing and record keeping that charges a fee to visit the farm, and record the weight of the milk produced by each goat, and other data such as milk fat, or reproductive performance. Using herd health analytical software, recommendations can be made, and genetic merit can be established.

The cost for milk testing can be prohibitive for some dairy goat producers, because there are no government subsidies or programs to offset the cost, compared to France and many other countries that have invested in the dairy goat sector. Participation is relatively more expensive for dairy goat producers because of the smaller volume of milk compared to cows. Dairy cow businesses can absorb the cost more easily because of their greater sales [24]. Still, use of DHIA is increasing, from less than 1% in 2004 to 13% of U.S. dairy goat herds in 2012.

### Prospects

There are two parallel dairy goat industries in the United States. The first is the industry begun in 1904 with small clubs to improve the genetics of breeds, such as Saanens or Nubians, which resulted in very high producing goats but often limited to small herds of purebred goats for local production. On the other hand, the commercial dairy goat industry is fast-growing but needs technical support for housing, herd management, feeding, genetics and reproduction [20]. Goat milk for cheese production is driving expansion of both groups, but the commercial herds are projected to become more dominant. U.S. goat cheeses can compete successfully, receiving multiple gold medals at international cheese competitions [25], which in turn attracts new consumers. Most US goat cheese is a soft curd cheese (“French style”), but the processors are innovative, and new types and flavors are appearing, such as Blueberry Lemon and Thyme Chèvre [20].

US dairy goat farms have become larger and more commercialized, but the majority are still small. In 2010, goat dairy operations averaged 11 goats per herd [25] and over 83% used hand milking. Milking machines are only found on the larger farms with enough milking animals to generate the income to pay for them. The larger farms are found close to goat milk processing plants, because transport costs are very high. Therefore, many smaller goat farms sell milk locally, or transform it to artisanal cheese or even goat milk soap, which are easier to store and transport.

In the United States and Canada, the main expenses of commercial dairy goat farms are feed costs, labor expenses and interest payments on debt. Profitability depends on keeping the cost of production (COP) low while developing new or strengthening existing markets. On farm feed production and increased mechanization can lower COP but often require large loans for the expansion. Compared to the dairy cow industry, which is characterized by significant investments in machinery, computers, and high-quality genetics, the U.S. dairy goat sector is considered “less mature”, because it is where the cow sector was about 15 years ago, regarding use of mechanization, computerized management, artificial insemination, marketing and specialized support from nutritionists, extension agents and veterinarians. Although outstanding breeding animals are available from US dairy herds, the commercial producers need help with genetic selection. The frozen semen or embryo sales tend to go to elite herds in the show circuit.

The mainstream dairy industry is starting to take note of the commercial potential of goats, with articles on dairy goat production now appearing in professional publications such as Progressive Dairyman, but technical information and specialized veterinary support can be hard to find in many areas. Goats in general, and dairy goats in particular, are considered a minor use species in the United States, and therefore few animal scientists or veterinarians have been trained in their peculiarities. For example, there are only 15 drugs labeled for goats, and most cannot be used in lactating animals [26].

Another important development in the dairy goat industry in the US is the growing strength of the organic market. Although good statistics are not available, there is increased interest in organic dairy goat products. The constraint is most likely the availability of land for the pasture requirement. This follows the trend in Europe, where the proportion of organic herds out of the total herds is much larger for goats than cows, suggesting greater dominance of the organic segment [27]. For example, the percentage of organic goat herds is 52.9% in Austria, 49% in Latvia, 31.5% in Estonia, 29.1% in the Czech Republic, 17.5% in Slovenia, 8.7% in Ireland, 7.5% in Italy, and 6.4% in the Netherlands. In Greece, which has the largest goat population in the EU, 4.1% of all goats are managed in organic systems, producing organic local cheese, such as feta. This trend will probably extend as consumers become more concerned and informed about their food choices.

If the goat dairy industry follows the dairy cow pattern, average production and efficiency will increase due to improved genetics, nutrition and management. In the US, the number of dairy cows decreased from just under 11 millions in 1985 to about 9 millions in 2018, while milk production per cow doubled during the same period. The contributors to this historical changes have been described [27].

## CANADA

Goat milk production in Canada has increased over the last decade with most of the growth coming from the eastern province of Ontario. There was a 79% increase in overall goat numbers from 2001 to 2016, from 183,000 to 230,074 head. Goat milk production increased 35% between 2006 to 2016, from 20.2 million liters to 57.4 million liters per year [28]. The majority of known dairy goat farms in Ontario had fewer than 1,000 goats in 2016. Those with fewer than 1,000 goats were also more profitable, with an expense-to-receipt ratio of 0.85, while the expense-to-receipt ratio of those reporting more than 1,000 goats was less favorable, at 0.88 [29].

Although the Canadian dairy goat sector is smaller than the US, it is expanding due to the same demand for good quality goat cheese by affluent urban consumers. National and provincial organizations are helping to organize the sector through education and research.

## GLOBALIZATION AND CONSOLIDATION

As the North American market for dairy goat products expands, and more money can be made in it, larger players want to become involved. Trade in products such as goat cheese, frozen curd for cheese making and dry milk is international, as are sales of frozen goat semen and embryos. The trade in baby formula based on goat milk is expanding rapidly, especially in Asia. There are wide variations around the world in the price paid to producers per tonne of goat milk, depending on the cost of inputs, the presence of subsidies as well as transport costs. Trade that is good for producers in one country can mean lost business in another. For example, cost differences means that a processor may prefer to import milk bought on the open market rather than buy from the nearest producer. Cost per tonne of goat milk in 2017 was reported to be $265, $738, and $958 in Mexico, Spain, and Canada, respectively [30].

Factories that produce goat cheese in the United States and Canada are increasingly consolidating, and some are becoming extremely large. The Canadian cheese manufacturer Saputo bought Woolwich, North America’s largest goat cheese manufacturer with two plants in Canada and one in Wisconsin (USA). In November 2017, Saputo announced it was acquiring Betin/Montchevre, which is Wisconsin’s largest goat cheese manufacturer. In California, the Swiss dairy cooperative Emmi has been buying several prominent goat cheese companies, including Cypress Grove Chevre, and Redwood Hill Farm. In January 2018, it bought Meyenberg Goat Milk Products, the only US producer of fluid goat milk that was sold in every state [20]. In 2017, the Chinese company Feihe announced plans to build an infant formula and milk powder plant in Ontario, Canada. The plan is to produce both cow milk and goat milk formula. The $225 million investment is the first of its kind in North America [31].

## BRAZIL

**Figure 4:**
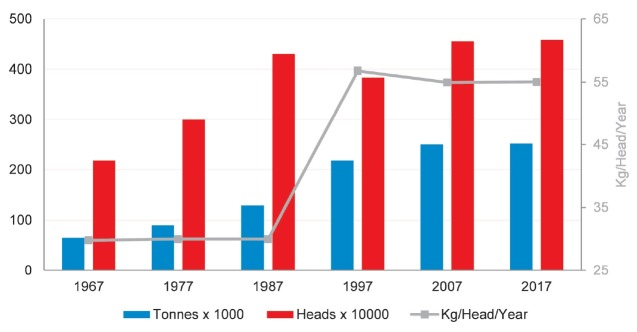
Trends of milk production from goats in Brazil (Compiled from [1], may include official, semi-official, estimated, or calculated data).

As the largest goat milk producing country in South America, Brazil produced over 250,000 tonnes of milk from goats in 2017, according the FAO estimate [1]. Since 1967, goat milk production has increased dramatically, tripling by 2017 (Figure 4). The production system is distinctively different in the North and South of the country. Northern and Northeastern Brazil, with close to 90% of total goat population of the country, is dominated by extensive production while more intensive operations can be found in Southern and Southeastern Brazil. Because of seasonal variation in rainfall, dairy goat producers often suffer from reduced milk production due to poor availability and nutritive value of forage during the dry season. Supplementation is essential to maintain milk production, but is not regularly practiced in Northern and Northeastern Brazil. The challenges are similar to those in Mexico. Production level is generally higher in Southern and Southeastern Brazil where most of the commercial producers, using European breeds and selling fluid milk, are concentrated.

The Brazilian Ministry of Agriculture initiated a Dairy Goat Breeding Plan in 2005, with Livestock and Food and the Association of Goats and Sheep Breeders of Minas Gerais. In testing more than 20 herds with Saanen, Alpine and Nubian bucks, the average total milk yield in a complete lactation was 768 kg, the 305 days milk yield was 676 kg, the lactation length was 278 days, and the daily yield was 2.75 kg/d from a total of approximately 8,000 tests [32]. While the production statistics were encouraging, the study also identified a number of opportunities, challenges and constraints including sustainability, organization, planning, selection traits, and market trends. Multivariate analyses were carried out to spatialize climatic, physical and socioeconomic variables in various dairy goat production systems in Brazil [33]. The highest yields of milk and goat production were observed in the Northeast manly due to larger animal numbers. The Southeast region was the second highest producer of milk, followed by the South, Midwest and North. It also revealed distinctions between clusters of political-administrative regions of Brazil. The climatic variables were the strongest contributions to the differences in dairy goat production between regions of Brazil.

Production and composition of goat milk in Brazil are influenced by genetic and non-genetic factors such as lactation order, herds, human index, and geographical location, with location the strongest determinant. Lactose levels were below the minimum limit established by the government, and SCCs averaged more than one million cells/mL [33]. However, when two dairy goat models were evaluated for income generation potential, it was concluded that it fit adequately into the household production model and could generate income competitively [34].

## ARGENTINA, BOLIVIA AND PERU

Dairy goat production is important in many parts of Argentina, for both economic and social reasons, because of increased demand for goat cheese in the urban areas. As in other parts of the Americas, a significant number of subsistence dairy goat producers are on marginal land that are not optimal for agricultural production. Most of Argentina’s goat milk is transformed into fermented products [35]. In the northern and central regions of Argentina, goat milk is mostly transformed into cheese, while in the La Pampa region, it is processed into powdered milk and ultra-high temperature fluid milk, in addition to cheese.

In Bolivia, most goats are found in marginal areas, the arid and semi-arid zones which lack infrastructure. As one of the lowest milk consumption countries in the world, an increase in goat milk production and consumption could improve infant and human nutrition and alleviate poverty. However, parents would need to supplement children at an early age. Bolivia produced 29,512 tonnes of goat milk in 2017, a 10% increase from 26,882 in 2010, the second year when the official data was recorded [1]. Bolivia’s organic agriculture has expanded in recent years mainly for export. Organic dairy goat production can build on that momentum to improve living standards of rural communities [36].

In Peru, goat milk production is estimated to be 23,750 tonnes in 2017 [1]. The statistics has not changed much over the years largely due to lack of accurate data collection. Peruvian goat production has suffered setbacks in the past due to cultural prejudice, scientific bias, bureaucratic politics and socio-economic conflicts [37]. The challenges and opportunities for dairy goat producers are expected to be similar to those in Bolivia, given very limited data available.

## CARIBBEAN

There are 3,516,607 goats in the countries of the Caribbean community (CARICOM), and the majority are found on small family farms practicing mixed, integrated crop-livestock farming [38]. Typically the small ruminants graze on land that is unsuitable for crop production, and use feed resources that cannot be consumed directly by humans. In Jamaica, Trinidad and Tobago, and Barbados, a small goat milk industry is developing using imported Alpine, Saanen, and Toggenburg breeds [39]. The dairy goat sector is much smaller than the meat goat sector in Trinidad and Tobago, and is small scale and low input. However, there has been an increase in the demand of goat milk as consumers are increasing aware of the nutrition merit of goat milk, creating an opportunity for producers. A well developed and revised “Dairy Goat Manual” was published by Trinidad and Tobago Goat and Sheep Society with the support of Interamerican Institute for Cooperation on Agriculture [40].

Goat milk production in Jamaica and Haiti is estimated to be 189,114 and 56,136 tonnes, respectively, based on imputation methodology [1]. There has been a renewed interest to further develop the dairy goat industry through dairy goat semen and embryo imports to Jamaica.

## PRODUCTION EFFICIENCY

**Figure 5:**
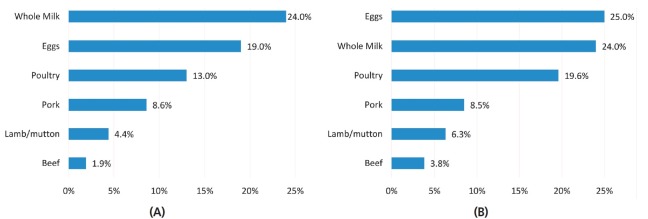
Energy (A) and protein (B) efficiencies of dairy and meat production: Data by [42]; artwork by OurWorldInData.org.

Because of human population growth, concern for resource utilization, and environmental impacts, all animal sectors are expected to produce more with less in the future. It will not be an exception for goats. In general, milk producing animals are more efficient in converting energy and protein to products than their counter parts (Figure 5). Like other milk producing animals, biological conversion of dairy goats is expected to be higher than those of meat animals due to daily milk production. One notable historical trend is the inverse relationship between number of dairy cows and the production in the United States [41]. The same trend in dairy goats can be observed during the past decade (2007 to 2017) when goat milk production increased by an average of 4.5% in the Americas and Europe while the dairy goat population decreased by nearly 1% [5].

In a natural environment, dairy goats can be efficient biological converters due to their unique eating behaviors [43,44] and more resistant to environmental stress [45]. With mobile lips, a prehensile tongue, agile front legs, and strong hind legs, goats are able to expand their feeding dimension and can employ desirable nutritional strategies. Selectivity, browsing, long distance traveling, bipedal stance, aerial positioning, and adaptability set goats apart from cattle and sheep in ingestion behaviors [44]. For subsistence dairy goat producers in the Americas, goats could better utilize nature resources that are not accessible to other dairy species.

## ENVIRONMENTAL IMPACT

Greenhouse gas emission and dairy waste disposal could be among the most important environmental concerns in dairy goat operations in the Americas. The link between dairy goat production and environment has been discussed separately [5]. The unique gastrointestinal tract, large populations, and appetite in ruminants result in global atmosphere emissions of mainly CO_2_, CH_4_, and N_2_O. These gases are considered anthropogenic, meaning that they result in environmental pollution caused by human activity. Globally, greenhouse gas emissions from goats and sheep are about 20% to 25% that of beef cattle and dairy cattle, with dairy goats contributing a significant amount among small ruminants [46]. In terms of unit of milk produced, small ruminants appear to emit more greenhouse gases than large ruminants [47]. However, in a small study with five farms [48], the average carbon footprint for indoor dairy goat farms (n = 3) was 11.05 t of CO_2_ e/ha and 0.81 kg of CO_2_ e/kg of fat and protein corrected milk (FPCM). For outdoor farms (n = 2), the average was 5.38 t of CO_2_ e/ha and 1.03 kg of CO_2_ e/kg of FPCM. These values are similar to those observed in dairy cows.

As a result of digestion and waste disposal, both CH_4_ and N_2_O are produced in dairy goats and N_2_O can contribute up to 18% of emissions [48]. Based on the Intergovernmental Panel on Climate Change Fourth Assessment Report (AR4), emissions of CH_4_ and N_2_O can be converted to CO_2_-eq by a factor of 25 and 298, respectively [49]. This underscores the importance of mitigation and abatement in all livestock species including dairy goats, for economic as well as environmental reasons. Effective recycling and utilization of manure also improves the environmental impact of dairy goat production. Effective manure management systems in dairy goat production can reduce the greenhouse gas emissions that plague our planet as discussed separately [5].

## ANIMAL WELFARE

Sensitivity towards the use of animals is increasing in the Americas, especially in prosperous urban areas. Ethological parameters encompass animal welfare, animal well-being, and human/animal interactions. There is general agreement that animals need to be free of thirst, hunger, and disease, for both production and the humanitarian considerations. On the other hand, production and management systems that affect animal response and behavior are fiercely debated among producers, consumers, and scientific community [27]. For example, some see an intensive system involving confinement negatively, because they disapprove of crowding and aggression, while others see it as positive because of freedom from harsh weather and parasites. Production systems, (mixed, intensive, extensive, and organic); housing (tie stalls, free stalls, and loose); and management systems (feeding, milking, processing animal waste, and flooring) can modify animal behavior and affect animal welfare. Access to pasture is increasingly viewed as a way to alleviate stress and to promote natural behavior in goats and other livestock species.

**Figure 6:**
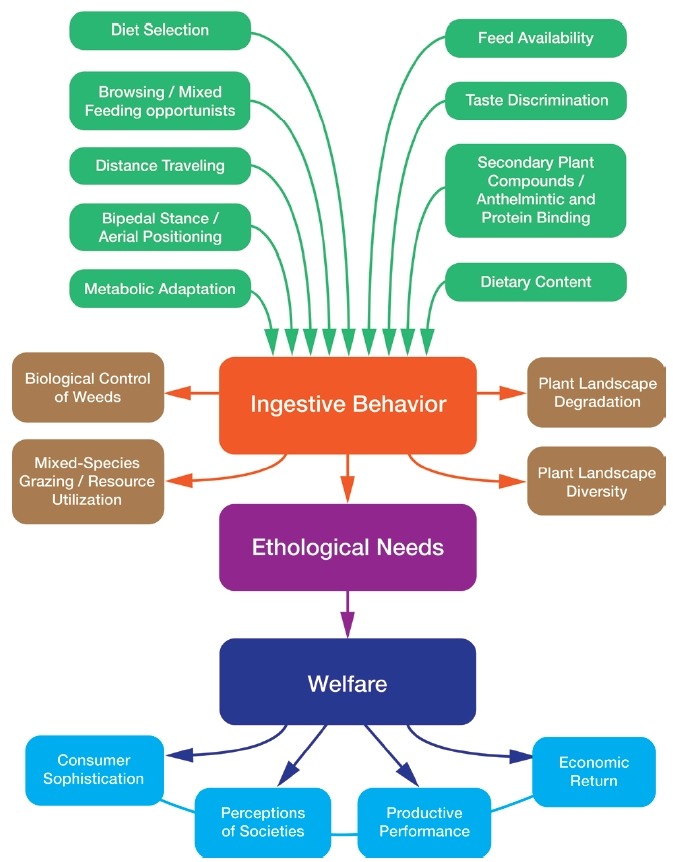
Ingestion behaviors, plant landscape, resource integration and animal welfare [44].

The scientific study of the unique behavior of goats in their usual environment becomes key to setting standards for welfare (Figure 6). Feeding systems must ensure that nutrient requirements are met, but also the accommodation of natural ingestion behaviors, wherever and whenever possible. Newer generations of consumers are increasingly sophisticated and willing to pay more for organic products, to promote environmental sustainability and animal welfare [14,15]. The welfare of farm animals is among the top three issues that European consumers want to know more about, after safety and quality of foods, and the effect of agriculture on environmental and climate change [50]. There is no reason to believe that consumers in the Americas are not moving in the same direction. While the debates continue, the cost of promoting natural goat behavior and living will have to be shared among stakeholders. As the market increasingly relies on commercial dairy goat production, often in confinement, it may be challenging to provide an environment that facilitates natural eating behavior. Subsistence dairy goat producers, on the other hand, provide a more natural environment, but have difficulty getting products to urban markets.

## CONCLUSION

The dairy goat industry in the countries of the Americas is smaller than the dairy cow sector but is poised for continued growth due to changing consumer preferences, government policies, climate change and need for alternative livelihoods. Because of the high biological conversion and production efficiency, dairy goats will play a vital role to supply products for human consumption. Both subsistence and commercial dairy goat operations exist in the Americas. In Northern Brazil, the Caribbean, and rural Mexico, dairy goat production is not only an important part of local culture but also is an integral part of the livelihoods for those living in marginal lands. In North America, goat cheese is an attractive product with desirable flavor and nutritional merit. Goat milk will continue to provide an alternative for those who are allergic to cow’s milk, but market growth is based on consumers who love goat cheese, and want to support local producers, who in turn must continue to improve product quality, variety and supply. Organic dairy production can be promising for producers on marginal lands but this requires significant organizing, training, management and integration into national and international markets. As the dairy goat industry continues to grow, animal welfare and environmental preservation must be addressed. Consumers are increasingly sophisticated and willing to pay a premium to know that animals are well-treated and can engage in natural behaviors.
